# Environmental signals driving liquid-liquid phase separation – a molecular memory in plants?

**DOI:** 10.3389/fpls.2024.1391043

**Published:** 2024-04-26

**Authors:** Ali Eljebbawi, Stephanie Hutin, Chloe Zubieta, Yvonne Stahl

**Affiliations:** ^1^ Institute for Developmental Genetics, Heinrich-Heine University, Düsseldorf, Germany; ^2^ Laboratoire de Physiologie Cellulaire et Végétale, Université Grenoble Alpes, Centre National de la Recherche Scientifique, Commissariat à l’énergie Atomique et aux Énergies Alternatives, Institut National de Recherche pour l’agriculture, l’alimentation et l’environnement, Institut de Recherche Interdisciplinaire de Grenoble, Grenoble, France

**Keywords:** liquid-liquid phase separation, condensates, abiotic stress, plant memory, environmental sensing

## Introduction

Plants face ever changing environmental conditions, where abiotic stresses such as temperature fluctuations, nutrient deficit, and hydric stress can impede their development, yield, and reproduction (van Wallendael et al., 2019; Lamers et al., 2020). Therefore, plants have evolved efficient adaptive mechanisms which boost their competence against recurring stresses. For example, exposure to extreme heat or cold can trigger adaptation through a priming process, leading to the formation of a “stress memory”, which consequently enhances the plant tolerance to these stresses during future encounters (Liu et al., 2022). In contrast, while low temperatures can act as a stress factor, they may also serve as a key developmental cue, particularly in the transition from vegetative to reproductive growth through vernalization. Unlike cold priming, vernalization exploits the memory of the prolonged winter cold to optimally time and induce flowering under warmer conditions (Sharma et al., 2022). These are examples of “long-term” memory, which are sustained by epigenetic mechanisms such as DNA methylation and histone modifications (Zhao et al., 2019; Liu and He, 2020). Remarkably, it can extend across generations via epigenetic inheritance, the activity of small RNAs, and chromatin remodeling (Molinier et al., 2006; Zhang and Tian, 2022). In addition, environmental memory can be “short-term”, temporarily persisting over one or more somatic mitotic divisions, and encompassing rapid and reversible changes in gene expression and hormonal signaling (Li et al., 2019; do Amaral et al., 2020). Similarly to long-term memory, this may be due to epigenetic mechanisms. However, recent studies suggest additional mechanisms for short-term memory, including protein phase separation.

Protein-driven LLPS is under active study as a mechanism for environmental sensing, response, and molecular memory in different organisms, including animals and yeast. During LLPS, proteins transition from a homogenous solution to distinct condensates (Musacchio, 2022), creating specialized environments for cellular processes (Chen et al., 2020; Maruri-López et al., 2021). For instance, LLPS is implicated in animal neuronal function, information processing, and memory storage in neuronal cells (Chen et al., 2020). Furthermore, P-bodies and stress granules, which regulate RNA metabolism and respond to cellular stress, are formed through LLPS, concentrating specific molecules for functions like mRNA degradation and translation repression, and are conserved across diverse eukaryotic organisms, including animal and yeast cells (Luo et al., 2018; Kearly et al., 2024). Also, the serotonin-induced aggregation of cytoplasmic polyadenylation element-binding proteins (CPEB) plays a role in long-term potentiation during courtship in Drosophila (Lau et al., 2020). In *Saccharomyces cerevisiae*, phase separation of WHI3, an RNA-binding protein, is involved in encoding memory of deceptive courtship (Lau et al., 2020). LLPS is also involved in chromatin organization and transcriptional regulation (Wang and Liu, 2019). To illustrate, condensates can bring together or isolate transcription factors (TFs) and coactivators or corepressors, creating and sustaining specific patterns of gene expression, which potentially influences cellular memory processes (Wang and Liu, 2019). While linking LLPS and memory remains an area of research and debate, exploring the roles of condensates in cellular organization, signaling, and gene regulation might offer valuable insights into cellular information storage. Although the putative role of LLPS in molecular memory has been primarily studied in yeast and animals, emerging evidence points to its role in plant stress and environmental memory.

Several pioneering studies demonstrate the significance of proteins undergoing LLPS in plant environmental response. In *Arabidopsis thaliana*, pivotal examples include the phase-separating proteins FRIGIDA (FRI), VERNALIZATION 1 (VRN1), and FLOWERING CONTROL LOCUS A (FCA), which collectively regulate *FLOWERING LOCUS C* (*FLC*) during vernalization, highlighting the role of LLPS in establishing a memory of past winter conditions to control flowering time (Levy et al., 2002; Fang et al., 2019; Zhou et al., 2019). Additionally, the thermosensory protein, EARLY FLOWERING 3 (ELF3), undergoes reversible phase separation with hysteresis behavior, potentially establishing a short-term memory of temperature (Jung et al., 2020; Murcia et al., 2022). In response to hyperosmotic stress, the transcriptional regulator SEUSS (SEU) undergoes condensation, where the formed condensates exhibit partial persistence, indicating the formation of a potential stress memory (Wang et al., 2022a). Moreover, FLOE1, a condensate-forming water sensor during seed germination, can form physiologically-relevant hydrogels, which may retain hydric stress memory (Dorone et al., 2021). This opinion aims to discuss the fundamental characteristics of LLPS in environmental sensing and explores the potential role of condensate formation as a molecular stress or environmental memory in plants.

## Main text

### Protein-mediated LLPS: a mechanism for changing cellular dynamics

Protein-mediated LLPS is highly dependent on protein concentration, ionic strength, pH, temperature, and the presence of different “clients” or “cargos”, such as other protein partners and/or nucleic acids (Elbaum-Garfinkle et al., 2015; Nott et al., 2015; Saha et al., 2016; Dignon et al., 2020). Proteins that drive LLPS are often characterized by intrinsically disordered regions (IDRs), which lack well-defined secondary and tertiary structure (Burke et al., 2015; Elbaum-Garfinkle et al., 2015; Kroschwald et al., 2018; Dignon et al., 2019). IDRs have a high degree of conformational flexibility and often exhibit multivalency, which is closely associated with the collective interactions between polypeptide chains (Pak et al., 2016). Accordingly, IDRs can drive phase separation via cation-pi, pi-pi, electrostatic attraction, and hydrophobic interactions (Tsang et al., 2020). It is worth noting that IDR-harboring proteins do not necessarily form condensates (Alberti et al., 2019). A subset of IDRs, called prion-like proteins or prion-like domains (PrD), are more frequently associated with phase separation, and are often enriched in asparagine and glutamine residues and bear sequence similar to yeast prion proteins. In addition to disordered proteins, LLPS can be initiated by folded domains such as coiled-coils (Ramšak et al., 2023)and via the formation of protein-RNA complexes mediated by RNA-recognition motifs (RRM) (Dignon et al., 2020; Ramšak et al., 2023).

Protein-mediated LLPS is commonly studied by diverse fluorescence-based techniques including Fluorescence Recovery After Photobleaching (FRAP) and Fluorescence Correlation Spectroscopy (FCS). FRAP is a method for determining the dynamics of fluorescently labelled proteins in a condensate. The fluorophore can be partially or fully photobleached and its fluorescence recovery curve examined. This analysis provides valuable insights into condensate dynamics, including the mobility and exchange of molecules within the condensates or between the condensate and the environment. Alternatively, FCS can analyze molecular dynamics in both *in vitro* and *in vivo* condensate phases, determining molecular concentration, oligomeric state, diffusion coefficient (hydrodynamic radius), and molecular interactions (Wang et al., 2022b). These techniques, while applicable in various research areas, are particularly instrumental in elucidating the behavior of biomolecular condensates, thus advancing our understanding of LLPS. Of note, based on FRAP and FCS experiments, many proteins, which initially form highly mobile liquid condensates, may become more visco-elastic and rigid as they “age” over time, forming gel-like states and fibrils (Molliex et al., 2015; Murakami et al., 2015; Patel et al., 2015; Wegmann et al., 2018; Ray et al., 2020). In animals, these fibrils have been linked to neurological diseases, including Alzheimer’s disease (Lin et al., 2015; Xiang et al., 2015; Pak et al., 2016). However, the physiological role of hydrogel/fibril formation in plants remains to be uncovered. However, the formation of gel and fibril states raises the intriguing question of whether or not this persistent state may act to establish a form of memory.

### Stable silencing of *FLOWERING LOCUS C* during vernalization

Environmental cues such as the prolonged exposure to low temperatures significantly influence the timing of flowering. The expression levels of the flowering repressor *FLC* time the onset of flowering in biennials and winter annual plants. The prolonged exposure to winter cold is required to repress *FLC* expression, allowing the plant to flower in the following spring. Vernalization, acting as an epigenetic memory of winter cold, determines the expression levels of *FLC*, synchronizing flowering to favorable spring conditions. High levels of *FLC* expression are maintained by the transcription factor FRI, resulting in suppression of flowering. Under low temperatures, the cold stabilization of FRI and its increased interaction with multiple factors drive the formation of FRI nuclear condensates, which requires its C-terminal IDR (Zhu et al., 2021). These nuclear condensates reduce FRI binding to the *FLC* locus via a sequestration mechanism, which consequently decreases *FLC* expression. Notably, FRI condensation is slowly reversible, dissipating within 5 hours upon returning to warmer conditions, suggesting that LLPS may act as a short-term memory of cold conditions (Zhu et al., 2021). Furthermore, noncoding RNAs may play a role in FRI LLPS, although there is some contradiction in the literature regarding their role in FRI condensation. Some evidence suggests that FRI condensation is promoted by cold-induced antisense RNA *COOLAIR* derived from the *FLC* locus (Zhu et al., 2021), while other studies indicate that condensation of FRI is independent of *COOLAIR* (Zhang et al., 2023).

**Figure 1:**
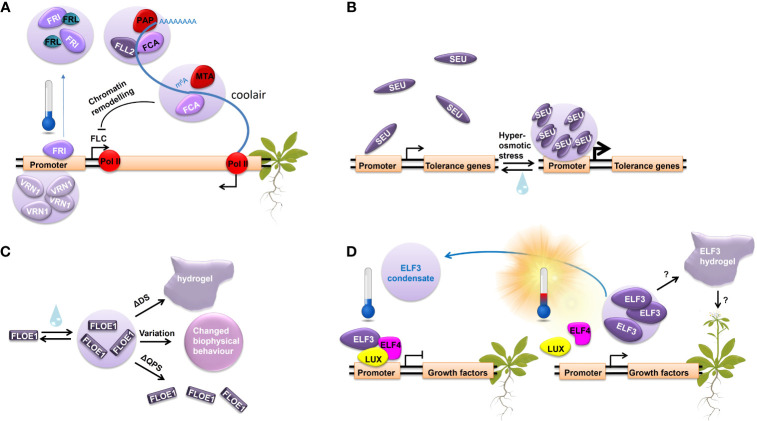
LLPS regulates important molecular processes in response to different stresses. **(A)** The molecular regulation of *FLC* repression during vernalization through LLPS. **(B)** SEUS LLPS in response to increasing extracellular osmolarity. **(C)** FLOE1 LLPS upon hydration governed by prion-like glutamine, proline, and serine rich (QPS).domain and the aspartic acid and serine rich (DS) domain, modulating its biological function. **(D)** ELF3 undergoes LLPS in response to temperature.

The RNA-binding protein FLOWERING CONTROL LOCUS A (FCA), containing two PrDs, and the coiled-coil protein, FLL2, promote nuclear body formation with *COOLAIR* and antagonize the activity of FRI, resulting in reduced *FLC* transcription (Fang et al., 2019) (**Figure 1A**). In addition, VERNALIZATION 1 (VRN1), a two B3 domain DNA-binding protein that binds non-specifically to DNA, undergoes LLPS and is required for the stable repression of *FLC* (Levy et al., 2002). VRN1 condensates form on the *FLC* locus, inducing structural changes in the *FLC* chromatin and ensuring *FLC* silencing (Zhou et al., 2019) (**Figure 1A**). In summary, several LLPS events elicit the stable repression of *FLC*, contributing to the molecular memory of winter cold. Notably, FRI condensation, which is cold-dependent and slowly dissipating upon a shift to warmer conditions, suggests that LLPS may act as a direct mechanism for cold memory.

### ELF3: a multivalent environmental sensor forming a short-term environmental memory

The transcriptional regulator ELF3 represents another example of LLPS in plant environmental response and possibly temperature memory establishment. ELF3 integrates information from the circadian clock, temperature, and light perception pathways to act as a key component in photoperiodic flowering (Ezer et al., 2017; Jung et al., 2020; Silva et al., 2020). It has no structured domains of known function and acts as a scaffold binding many TFs (Hicks et al., 2001; Nusinow et al., 2011; Herrero et al., 2012; Ezer et al., 2017; Silva et al., 2020). Upon exposure to higher ambient temperature, ELF3 undergoes reversible LLPS driven by its C-terminal PrD and fine-tuned by the length of polyglutamine regions in this domain (7 to 29 glutamines across 181 natural *A. thaliana* accessions) (Jung et al., 2020; Hutin et al., 2023). *In vitro* and *in vivo* FRAP experiments on ELF3 condensates demonstrate an aging process in which a low mobility hydrogel forms. In addition, *in vitro* temperature ramp experiments for ELF3 PrD reveal a delay in phase separation reversibility upon the return to lower temperatures, indicating a degree of hysteresis in the protein behavior (Hutin et al., 2023). This suggests that the system may maintain a memory of its past states. Indeed, *in planta* ELF3 forms nuclear condensates in response to increasing temperatures (**Figure 1D**). Their reversibility via cooling requires a more pronounced temperature shift or longer time compared to the warm temperature-induced LLPS. Consequently, as ELF3 remains in the inactive phase separated state, the regulation of its target genes is delayed, thereby potentially establishing a short-term memory of temperature (Murcia et al., 2022).

### SEUSS and FLOE1: response to osmotic stress and water availability

In response to hyperosmotic stress, the transcriptional regulator SEU forms condensates due to conformational changes within its N-terminal IDR. SEU condensation, which likely occurs via LLPS, is triggered by intracellular molecular crowding. It is vital for the stress response and the subsequent expression of osmotic stress-responsive genes (Wang et al., 2022a) (**Figure 1B**). Although SEU forms reversable condensates, FRAP analysis revealed their incomplete recovery, suggesting that they exhibit partial persistence with lower mobility or reduced turnover. This incomplete recovery hints at the possibility that SEU forms a gel-like state, indicating a potential mechanism for retaining a stress memory.

Additionally, FLOE1 is an PrD-containing protein that suppresses seed germination under unfavorable conditions (Dorone et al., 2021). It undergoes LLPS upon hydration, allowing the embryo to sense water (**Figure 1C**). Interestingly, its biological function is modulated by the biophysical state of its condensates, which is governed by the glutamine, proline, and serine rich (QPS) domain and the aspartic acid and serine rich (DS) domain. While the QPS domain is responsible for driving FLOE1 condensation and its function in water-sensing, the DS domain regulates the fluidity of its condensates. Natural variation in the DS domain across ecotypes promotes local adaptation by fine-tuning the fluidity of FLOE1 condensates (Dorone et al., 2021). Notably, FLOE1 condensates appear spontaneously and exhibit full reversibility through repeated hydration-dehydration cycles. However, deleting the DS domain leads to the formation of an irreversible hydrogel and promotes germination under stress, indicating that changes in the dynamics of FLOE1 condensates affect the stress response (Dorone et al., 2021).

## Concluding remarks

Exploring protein-mediated LLPS in plant cells highlights its intriguing roles in cellular dynamics, stress perception, and potentially, environmental or stress memory. In other organisms, memory effects of protein phase separation are better established. For example, in yeast, prion proteins provide a nongenetic method of inheritance and may provide an adaptive advantage in different environments and under different stress conditions (Chakrabortee et al., 2016). The self-replicating structures of prion proteins separate genotype from phenotype, enabling genetically identical cells within a population to adopt new traits (Byers and Jarosz, 2014). In mammalian systems, the neuronal protein, CaMKII, involved in synaptic plasticity, undergoes phase separation triggered by Ca^2+^ and this state persists even after the Ca^2+^ is removed, maintaining a memory of the calcium signal (Hosokawa et al., 2021). The sensitivity of condensate formation to various physical factors suggests that abiotic stresses may be detected by LLPS in plants (Burkart et al., 2022). Although confirming the role of these condensates in establishing environmental memory in plants is in its nascency, there is intriguing preliminary evidence pointing to the involvement of phase separation in this process. Potential mechanisms of LLPS mediated short-term memory might include changes in protein dynamics, hysteresis of condensate reversibility and irreversible gel formation. The ability of condensates to adopt different states (liquid, gel and/or fibril) raise intriguing questions about the persistence and functional implications of these condensates in plant environmental memory. Further investigations into the structural, biomechanical, and biochemical basis of protein phase separation in plant physiology will expand our understanding of this dynamic cellular process and whether this is a mechanism plants use to establish a physiological “memory” of past environmental events.

## Author contributions

AE: Conceptualization, Writing – original draft, Writing – review & editing. SH: Visualization, Writing – original draft, Writing – review & editing. CZ: Writing – review & editing. YS: Conceptualization, Funding acquisition, Project administration, Writing – review & editing.

## References

[B1] Alberti S. Gladfelter A. Mittag T. (2019). Considerations and challenges in studying liquid-liquid phase separation and biomolecular condensates. Cell 176, 419–434. doi: 10.1016/j.cell.2018.12.035 30682370 PMC6445271

[B2] Burkart R. C. Eljebbawi A. Stahl Y. (2022). Come together now: Dynamic body-formation of key regulators integrates environmental cues in plant development. Front. Plant Sci. 13. doi: 10.3389/fpls.2022.1052107 PMC9702078 36452084

[B3] Burke K. A. Janke A. M. Rhine C. L. Fawzi N. L. (2015). Residue-by-residue view of *in vitro* FUS granules that bind the C-terminal domain of RNA polymerase II. Mol. Cell 60, 231–241. doi: 10.1016/j.molcel.2015.09.006 26455390 PMC4609301

[B4] Byers J. S. Jarosz D. F. (2014). Pernicious pathogens or expedient elements of inheritance: the significance of yeast prions. PloS Pathog. 10, e1003992. doi: 10.1371/journal.ppat.1003992 24722628 PMC3983059

[B5] Chakrabortee S. Byers J. S. Jones S. Garcia D. M. Bhullar B. Chang A.. (2016). Intrinsically disordered proteins drive emergence and inheritance of biological traits. Cell 167, 369–381.e12. doi: 10.1016/j.cell.2016.09.017 27693355 PMC5066306

[B6] Chen X. Wu X. Wu H. Zhang M. (2020). Phase separation at the synapse. Nat. Neurosci. 23, 301–310. doi: 10.1038/s41593-019-0579-9 32015539

[B7] Dignon G. L. Best R. B. Mittal J. (2020). Biomolecular phase separation: from molecular driving forces to macroscopic properties. Annu. Rev. Phys. Chem. 71, 53–75. doi: 10.1146/annurev-physchem-071819-113553 32312191 PMC7469089

[B8] Dignon G. L. Zheng W. Kim Y. C. Mittal J. (2019). Temperature-controlled liquid-liquid phase separation of disordered proteins. ACS Cent. Sci. 5, 821–830. doi: 10.1021/acscentsci.9b00102 31139718 PMC6535772

[B9] do Amaral M. N. Arge L. W. P. Auler P. A. Rossatto T. Milech C. de Magalhães A. M.. (2020). Long-term transcriptional memory in rice plants submitted to salt shock. Planta 251, 111. doi: 10.1007/s00425-020-03397-z 32474838

[B10] Dorone Y. Boeynaems S. Flores E. Jin B. Hateley S. Bossi F.. (2021). A prion-like protein regulator of seed germination undergoes hydration-dependent phase separation. Cell 184, 4284–4298.e27. doi: 10.1016/j.cell.2021.06.009 34233164 PMC8513799

[B11] Elbaum-Garfinkle S. Kim Y. Szczepaniak K. Chen C. C.-H. Eckmann C. R. Myong S.. (2015). The disordered P granule protein LAF-1 drives phase separation into droplets with tunable viscosity and dynamics. Proc. Natl. Acad. Sci. United States America 112, 7189–7194. doi: 10.1073/pnas.1504822112 PMC4466716 26015579

[B12] Ezer D. Jung J.-H. Lan H. Biswas S. Gregoire L. Box M. S.. (2017). The evening complex coordinates environmental and endogenous signals in Arabidopsis. Nat. Plants 3, 17087. doi: 10.1038/nplants.2017.87 28650433 PMC5495178

[B13] Fang X. Wang L. Ishikawa R. Li Y. Fiedler M. Liu F.. (2019). Arabidopsis FLL2 promotes liquid-liquid phase separation of polyadenylation complexes. Nature 569, 265–269. doi: 10.1038/s41586-019-1165-8 31043738 PMC6625965

[B14] Herrero E. Kolmos E. Bujdoso N. Yuan Y. Wang M. Berns M. C.. (2012). EARLY FLOWERING4 recruitment of EARLY FLOWERING3 in the nucleus sustains the Arabidopsis circadian clock. Plant Cell 24, 428–443. doi: 10.1105/tpc.111.093807 22327739 PMC3315225

[B15] Hicks K. A. Albertson T. M. Wagner D. R. (2001). EARLY FLOWERING3 encodes a novel protein that regulates circadian clock function and flowering in Arabidopsis. In Plant Cell 13, 1281–1292. doi: 10.1105/TPC.010070 PMC135582 11402160

[B16] Hosokawa T. Liu P.-W. Cai Q. Ferreira J. S. Levet F. Butler C.. (2021). CaMKII activation persistently segregates postsynaptic proteins via liquid phase separation. Nat. Neurosci. 24, 777–785. doi: 10.1038/s41593-021-00843-3 33927400

[B17] Hutin S. Kumita J. R. Strotmann V. I. Dolata A. Ling W. L. Louafi N.. (2023). Phase separation and molecular ordering of the prion-like domain of the Arabidopsis thermosensory protein EARLY FLOWERING 3. Proc. Natl. Acad. Sci. United States America 120, e2304714120. doi: 10.1073/pnas.2304714120 PMC10334799 37399408

[B18] Jung J.-H. Barbosa A. D. Hutin S. Kumita J. R. Gao M. Derwort D.. (2020). A prion-like domain in ELF3 functions as a thermosensor in Arabidopsis. Nature 585, 256–260. doi: 10.1038/s41586-020-2644-7 32848244

[B19] Kearly A. Nelson A. D.L. Skirycz A. Chodasiewicz M. (2024). Composition and function of stress granules and P-bodies in plants. Semin. Cell Dev. Biol. 156, 167–175. doi: 10.1016/j.semcdb.2022.11.008 36464613

[B20] Kroschwald S. Munder M. C. Maharana S. Franzmann T. M. Richter D. Ruer M.. (2018). Different material states of pub1 condensates define distinct modes of stress adaptation and recovery. Cell Rep. 23, 3327–3339. doi: 10.1016/j.celrep.2018.05.041 29898402

[B21] Lamers J. van der Meer T. Testerink C. (2020). How plants sense and respond to stressful environments. Plant Physiol. 182, 1624–1635. doi: 10.1104/pp.19.01464 32132112 PMC7140927

[B22] Lau Y. Oamen H. P. Caudron F. (2020). Protein phase separation during stress adaptation and cellular memory. Cells 9, (5). doi: 10.3390/cells9051302 PMC7291175 32456195

[B23] Levy Y. Y. Mesnage S. Mylne J. S. Gendall A. R. Dean C. (2002). Multiple roles of Arabidopsis VRN1 in vernalization and flowering time control. Sci. (New York N.Y.) 297, 243–246. doi: 10.1126/science.1072147 12114624

[B24] Li P. Yang H. Wang L. Liu H. Huo H. Zhang C.. (2019). Physiological and transcriptome analyses reveal short-term responses and formation of memory under drought stress in rice. Front. Genet. 10, 55. doi: 10.3389/fgene.2019.00055 30800142 PMC6375884

[B25] Lin Y. Protter D. S.W. Rosen M. K. Parker R. (2015). Formation and maturation of phase-separated liquid droplets by RNA-binding proteins. Mol. Cell 60, 208–219. doi: 10.1016/j.molcel.2015.08.018 26412307 PMC4609299

[B26] Liu H. Able A. J. Able J. A. (2022). Priming crops for the future: rewiring stress memory. Trends Plant Sci. 27, 699–716. doi: 10.1016/j.tplants.2021.11.015 34906381

[B27] Liu J. He Z. (2020). Small DNA methylation, big player in plant abiotic stress responses and memory. Front. Plant Sci. 11. doi: 10.3389/fpls.2020.595603 PMC7758401 33362826

[B28] Luo Y. Na Z. Slavoff S. A. (2018). P-bodies: composition, properties, and functions. Biochemistry 57, 2424–2431. doi: 10.1021/acs.biochem.7b01162 29381060 PMC6296482

[B29] Maruri-López I. Figueroa N. E. Hernández-Sánchez I. E. Chodasiewicz M. (2021). Plant stress granules: trends and beyond. Front. Plant Sci. 12. doi: 10.3389/fpls.2021.722643 PMC8381727 34434210

[B30] Molinier J. Ries G. Zipfel C. Hohn B. (2006). Transgeneration memory of stress in plants. Nature 442, 1046–1049. doi: 10.1038/nature05022 16892047

[B31] Molliex A. Temirov J. Lee J. Coughlin M. Kanagaraj A. P. Kim H. J.. (2015). Phase separation by low complexity domains promotes stress granule assembly and drives pathological fibrillization. Cell 163, 123–133. doi: 10.1016/j.cell.2015.09.015 26406374 PMC5149108

[B32] Murakami T. Qamar S. Lin J. Q. Schierle G. S. Kaminski R. E. Miyashita A.. (2015). ALS/FTD mutation-induced phase transition of FUS liquid droplets and reversible hydrogels into irreversible hydrogels impairs RNP granule function. Neuron 88, 678–690. doi: 10.1016/j.neuron.2015.10.030 26526393 PMC4660210

[B33] Murcia G. Nieto C. Sellaro R. Prat S. Casal J. J. (2022). Hysteresis in PHYTOCHROME-INTERACTING FACTOR 4 and EARLY-FLOWERING 3 dynamics dominates warm daytime memory in Arabidopsis. Plant Cell 34, 2188–2204. doi: 10.1093/plcell/koac078 35234947 PMC9134080

[B34] Musacchio A. (2022). On the role of phase separation in the biogenesis of membraneless compartments. EMBO J. 41, e109952. doi: 10.15252/embj.2021109952 35107832 PMC8886532

[B35] Nott T. J. Petsalaki E. Farber P. Jervis D. Fussner E. Plochowietz A.. (2015). Phase transition of a disordered nuage protein generates environmentally responsive membraneless organelles. Mol. Cell 57, 936–947. doi: 10.1016/j.molcel.2015.01.013 25747659 PMC4352761

[B36] Nusinow D. A. Helfer A. Hamilton E. E. King J. J. Imaizumi T. Schultz T. F.. (2011). The ELF4-ELF3-LUX complex links the circadian clock to diurnal control of hypocotyl growth. Nature 475, 398–402. doi: 10.1038/nature10182 21753751 PMC3155984

[B37] Pak C. W. Kosno M. Holehouse A. S. Padrick S. B. Mittal A. Ali R.. (2016). Sequence determinants of intracellular phase separation by complex coacervation of a disordered protein. Mol. Cell 63, 72–85. doi: 10.1016/j.molcel.2016.05.042 27392146 PMC4973464

[B38] Patel A. Lee H. O. Jawerth L. Maharana S. Jahnel M. Hein M. Y.. (2015). A liquid-to-solid phase transition of the ALS protein FUS accelerated by disease mutation. Cell 162, 1066–1077. doi: 10.1016/j.cell.2015.07.047 26317470

[B39] Ramšak M. Ramirez D. A. Hough L. E. Shirts M. R. Vidmar S. Eleršič Filipič K.. (2023). Programmable *de novo* designed coiled coil-mediated phase separation in mammalian cells. Nat. Commun. 14, 7973. doi: 10.1038/s41467-023-43742-w 38042897 PMC10693550

[B40] Ray S. Singh N. Kumar R. Patel K. Pandey S. Datta D.. (2020). α-Synuclein aggregation nucleates through liquid-liquid phase separation. Nat. Chem. 12, 705–716. doi: 10.1038/s41557-020-0465-9 32514159

[B41] Saha S. Weber C. A. Nousch M. Adame-Arana O. Hoege C. Hein M. Y.. (2016). Polar positioning of phase-separated liquid compartments in cells regulated by an mRNA competition mechanism. Cell 166, 1572–1584.e16. doi: 10.1016/j.cell.2016.08.006 27594427 PMC5034880

[B42] Sharma M. Kumar P. Verma V. Sharma R. Bhargava B. Irfan M. (2022). Understanding plant stress memory response for abiotic stress resilience: Molecular insights and prospects. Plant Physiol. Biochem. PPB 179, 10–24. doi: 10.1016/j.plaphy.2022.03.004 35305363

[B43] Silva C. S. Nayak A. Lai X. Hutin S. Hugouvieux V. Jung J.-H.. (2020). Molecular mechanisms of Evening Complex activity in Arabidopsis. Proc. Natl. Acad. Sci. United States America 117, 6901–6909. doi: 10.1073/pnas.1920972117 PMC7104408 32165537

[B44] Tsang B. Pritišanac I. Scherer S. W. Moses A. M. Forman-Kay J. D. (2020). Phase separation as a missing mechanism for interpretation of disease mutations. Cell 183, 1742–1756. doi: 10.1016/j.cell.2020.11.050 33357399

[B45] van Wallendael A. Soltani A. Emery N. C. Peixoto M. M. Olsen J. Lowry D. B. (2019). A molecular view of plant local adaptation: incorporating stress-response networks. Annu. Rev. Plant Biol. 70, 559–583. doi: 10.1146/annurev-arplant-050718-100114 30786237

[B46] Wang N. Liu C. (2019). Implications of liquid-liquid phase separation in plant chromatin organization and transcriptional control. Curr. Opin. Genet. Dev. 55, 59–65. doi: 10.1016/j.gde.2019.06.003 31306885

[B47] Wang B. Zhang H. Huai J. Peng F. Wu J. Lin R.. (2022a). Condensation of SEUSS promotes hyperosmotic stress tolerance in Arabidopsis. Nat. Chem. Biol. 18, 1361–1369. doi: 10.1038/s41589-022-01196-z 36376475

[B48] Wang Z. Zhang H. Jian L. Ding B. Huang K. Zhang W.. (2022b). Principles of fluorescence correlation spectroscopy applied to studies of biomolecular liquid-liquid phase separation. swwlxb 8, 100–118. doi: 10.52601/bpr.2022.210047 PMC10195812 37287826

[B49] Wegmann S. Eftekharzadeh B. Tepper K. Zoltowska K. M. Bennett R. E. Dujardin S.. (2018). Tau protein liquid-liquid phase separation can initiate tau aggregation. EMBO J. 37. doi: 10.15252/embj.201798049 PMC5881631 29472250

[B50] Xiang S. Kato M. Wu L. C. Lin Y. Ding M. Zhang Y.. (2015). The LC domain of hnRNPA2 adopts similar conformations in hydrogel polymers, liquid-like droplets, and nuclei. Cell 163, 829–839. doi: 10.1016/j.cell.2015.10.040 26544936 PMC4879888

[B51] Zhang Z. Luo X. Yang Y. He Y. (2023). Cold induction of nuclear FRIGIDA condensation in Arabidopsis. Nature 619, E27–E32. doi: 10.1038/s41586-023-06189-z 37438599 PMC10338335

[B52] Zhang Q. Tian Y. (2022). Molecular insights into the transgenerational inheritance of stress memory. J. Genet. Genomics 49, 89–95. doi: 10.1016/j.jgg.2021.11.015 34923165

[B53] Zhao T. Zhan Z. Jiang D. (2019). Histone modifications and their regulatory roles in plant development and environmental memory. J. Genet. Genomics = Yi Chuan xue bao 46, 467–476. doi: 10.1016/j.jgg.2019.09.005 31813758

[B54] Zhou H. Song Z. Zhong S. Zuo L. Qi Z. Qu L.-J.. (2019). Mechanism of DNA-induced phase separation for transcriptional repressor VRN1. Angewandte Chemie 131, 4912–4916. doi: 10.1002/ange.201810373 30762296

[B55] Zhu P. Lister C. Dean C. (2021). : Cold-induced Arabidopsis FRIGIDA nuclear condensates for FLC repression. Nature 599, 657–661. doi: 10.1038/s41586-021-04062-5 34732891 PMC8612926

